# Current-induced Orbital and Spin Magnetizations in Crystals with Helical Structure

**DOI:** 10.1038/srep12024

**Published:** 2015-07-09

**Authors:** Taiki Yoda, Takehito Yokoyama, Shuichi Murakami

**Affiliations:** 1 Department of Physics, Tokyo Institute of Technology, Tokyo 152-8551, Japan; 2 TIES, Tokyo Institute of Technology, 2-12-1 Ookayama, Meguro-ku, Tokyo 152-8551, Japan

## Abstract

We theoretically show that in a crystal with a helical lattice structure, orbital and spin magnetizations along a helical axis are induced by an electric current along the helical axis. We propose a simple tight-binding model for calculations, and the results can be generalized to any helical crystals. The induced magnetizations are opposite for right-handed and left-handed helices. The current-induced spin magnetization along the helical axis comes from a radial spin texture on the Fermi surface. This is in sharp contrast to Rashba systems where the induced spin magnetization is perpendicular to the applied current.

Recent discovery of novel physics due to an interplay between electricity and magnetism such as multiferroics^1^,^2^,^3^,^4^, skyrmion^5^,^6^,^7^,^8^, and current-induced magnetization reversal^9^,^10^,^11^,^12^, makes it possible to control a magnetization with electric field. However, there is a well-known classical example; an electric current flowing through a solenoid induces a magnetization. In this paper, we theoretically propose a condensed-matter analogue of a solenoid. We consider a three-dimensional crystal with a helical lattice structure. For crystals with helical crystal structure such as Se or Te, one can define handedness similar to a solenoid. Keeping these crystals in mind, we propose a simple tight-binding model with handedness, which can grasp the essence of the physics of helical crystals. Then we show that orbital and spin magnetization along a helical axis is induced by an electric current along the helical axis. The induced magnetizations are opposite for right-handed and left-handed helices. In contrast to Rashba system, the spin texture on the Fermi surface is radial, and the current-induced spin magnetization along the helical axis in helical crystals is attributed to this radial spin texture. Our results can be generalized to any crystals without mirror and inversion symmetries and they would pave the way to spintronics application of helical crystals.

First, we consider an orbital magnetization in a helical crystal. Here, we introduce a three-dimensional tight-binding model with a right- or left-handed helical structures. The lattice structure of this model is composed of an infinite stack of honeycomb lattice layers with one orbital per site. We consider the honeycomb lattice as shown in Fig.1a, with 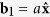, 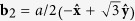, and **b**_3_ = −**b**_1_ − **b**_2_, where *a* is a constant. The layers are stacked along the *z*-direction with the primitive lattice vector 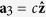, where *c* is the interlayer spacing. The Hamiltonian is


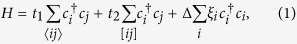


where *t*_1_, *t*_2_ and Δ are real and we set *t*_1_ > 0 for simplicity. The first term is a nearest-neighbor hopping term within the *xy* plane. The second term represents “helical” hoppings between the same sublattice in the neighboring layers. This term is different between a right-handed and a left-handed helices as shown in Fig.1b,c. In the right-handed helix (Fig.1b), the direction of hoppings between A sites are 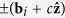, and that between B sites are 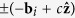. Similarly in the left-handed helix (Fig.1c) the direction of hoppings between A sites are 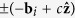, and that between B sites are 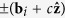. This term breaks inversion and mirror symmetries. The third term is a staggered on-site potential, where *ξ*_*i*_ is +1 and −1 for the sites in A and B sublattices, respectively. The space group of our model is *P*321, with *C*_2*y*_ and *C*_3*z*_ symmetries. Only when Δ = 0, the space group becomes *P*622, having additional *C*_2*x*_ and *C*_6*z*_ symmetries.

We note that the Hamiltonian for the right-handed (left-handed) helix becomes the Haldane model on a honeycomb lattice^13^ by replacing +*k*_*z*_*c* (−*k*_*z*_*c*) with a Aharonov-Bohm phase *ϕ* in the second-neighbor hopping in the Haldane model. We also note that the time-reversal symmetry is present in our model, while it is absent in the Haldane model for *ϕ* ≠ 0. Hence the band structure of our model is easily obtained from that of the Haldane model^13^. The Brillouin zone (BZ) and band structure of our model are shown in Fig. 2a–c, respectively. In the BZ, the K (K’) point is defined by **k** ⋅ **b**_*i*_ = −2*π*/3 (2*π*/3) on the *k*_*z*_ = 0 plane, and the H (H’) point is similarly defined on the *k*_*z*_ = *π*/*c* plane.

Using this model, we calculate the orbital magnetization induced by an electric field along the helical axis. In the limit of zero temperature *T* → 0, it is calculated from the formula^14^,^15^,^16^,^17^,^18^,^19^,^20^


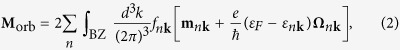


where the integral is performed over the BZ, *n* denotes the band index, *f*_*n***k**_ is the distribution function for the eigenenergy *ε*_*n***k**_, *ε*_*F*_ is the Fermi energy, and the prefactor 2 comes from the spin degeneracy. The orbital magnetic moment of the Bloch electrons is defined by^21^,^22^ 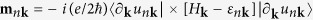 and the Berry curvature is defined by 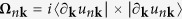, where 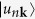 is the periodic part of the Bloch function. Because our model lacks inversion symmetry, both **m**_*n***k**_ and Ω_*n***k**_ are allowed to have nonzero values for any **k**. However, in equilibrium, due to the time-reversal symmetry **M**_orb_ is zero because in Eq. (2) the contribution from **k** and that from −**k** cancel each other. To induce the orbital magnetization, we apply an electric field along the *z* axis. For a metal it induces a charge current accompanied by nonequilibrium electron distribution, and orbital magnetization is expected to arise. Within the Boltzmann approximation, the applied electric field *E*_*z*_ changes *f*_*n***k**_ into


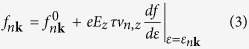


where 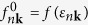 is the Fermi distribution function in equilibrium, *τ* is the relaxation time assumed to be constant, and *v*_*n*,*z*_ = (1/*ħ*)∂*ε*_*n***k**_/∂*k*_*z*_ is the velocity in the *z* direction. Substituting *f*_*n***k**_ into Eq. (2), we obtain the current-induced orbital magnetization **M**_orb_, which is along the *z*-axis by symmetry. Because of time-reversal symmetry, the 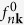 term in Eq. (3) does not contribute to **M**_orb_. In the *T* → 0 limit, ∂*f*/∂*ε* has a sharp peak at *ε*_*F*_. Therefore, for a band insulator, the induced orbital magnetization **M**_orb_ is zero. This is in contrast to multiferroics. Furthermore, the Berry-curvature term (second term in Eq. (2)) always vanishes at *T* → 0.

Figure 2d,e shows numerical results of Eq. (2) for several values of Δ. We set the parameters as *t*_2_ = *t*_1_/3. The band structure is easily obtained from the similarlity to the Haldane model^13^. For |Δ| > 2*t*_1_, the bands have a finite gap. For 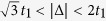, the two bands overlap and the system is in the semimetal phase. For 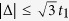, the two bands cross each other only on K-H line and K’-H’ line at 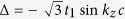 and 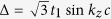, forming Dirac cones at energies 
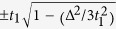
, respectively. As is expected, when the Fermi energy lies in the energy gap (Δ = 3*t*_1_, −*t*_1_ < *ε*_*F*_ < *t*_1_ in Fig.2d,e), the orbital magnetization is zero for *T* → 0. For metals (Δ = 0 and Δ = *t*_1_), the current-induced orbital magnetization appears. In this case, the induced magnetization is largely enhanced around *ε*_*F*_ ≃ *t*_1_. This is attributed to an enhanced orbital magnetic moment **m**_*n***k**_ near the Dirac points appearing on the K-H or K’-H’ lines. The orbital magnetization in the left-handed helix (Fig.2e) is exactly opposite to that in the right-handed one (Fig.2d). This dependence on handedness is similar to the solenoid, where an electric current generates a magnetic field. Thus, based on the semiclassical theory, the orbital magnetization induced by the current is attributed to a helical motion of a wavepacket. Here, we note that although most of the magnetoelectric effect involve the spin-orbit interaction, the current-induced orbital magnetization predicted here appears even without the spin-orbit interaction.

Next, we consider current-induced spin polarization. To this end, we introduce the spin-orbit interaction into a tight-binding model with the helical structure:


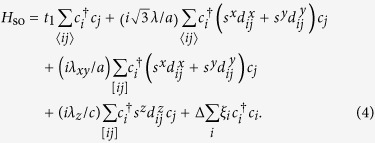


The first term is a spin-independent nearest-neighbor hopping term within the *xy* plane and the fifth term is a staggered on-site potential term. The other three terms represent spin-orbit interactions (*s* are the Pauli matrices in spin space). The second term is a spin-dependent nearest-neighbor hopping term within the *xy* plane. The third and fourth terms involve spin-dependent helical hoppings between the neighboring layers as shown in Fig.1b,c, where 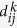 is the *k*-component of a vector pointing from site *j* to site *i*. The space group of this model is the same as that of the model (1).

Figure 3a shows the band structure of the Hamiltonian (4) along some high symmetry lines. Figure 3b shows the spin texture projected onto the *xy* plane, on the Fermi surface around the H point at *ε*_*F*_ = 0.68*t*_1_. In fact, the spin around the H point has not only the *xy*-component but also the *z*-component. Because of the spin-orbit interaction, the two spin-split Fermi surfaces appear, having the opposite spin orientations. Remarkably, unlike Rashba systems, the spin is oriented radially, and rotates once around the H point. Figure 3c shows the spin texture between the K and H points at *ε*_*F*_ = 0.68*t*_1_. In this case, one of the spin-split Fermi surface is open. Nevertheless, the inner Fermi surface have a radial spin texture. This spin texture results from crystal symmetries. Namely, the K-H lines are three-fold rotation axis and hence the spin on these lines are parallel to these K-H lines. Furthermore, the absence of mirror symmetries is crucial for the radial spin textures; if a mirror plane including the *xy* plane or the *z* axis were present, the spins should be perpendicular to the mirror plane, and a radial spin texture would not appear. For example, Te and Se have helical crystal structure, and as expected, they have been predicted to show a radial spin texture^23^.

We now calculate current-induced spin magnetization in the present system. It is known that in Rashba systems the current-induced magnetization is perpendicular to the current, because of the tangential spin texture in the spin-split Fermi surfaces^24^,^25^,^26^,^27^. Similarly, a spin magnetization is induced by an electric current in our model, whereas the magnetization is parallel to the current when the current is along the helical axis by symmetry, thanks to the radial spin texture. Within the Boltzmann approximation as explained in the Methods section, the *z*-component of **M**_spin_ for several parameters is numerically calculated as shown in Fig. 3d. We fix *λ*_*xy*_ = 0.05*t*_1_, *λ*_*z*_ = 0.05*t*_1_, and Δ = 0.4*t*_1_. As is expected, for a metal, the spin magnetization parallel to the current is induced by the current. Similar to the orbital magnetization, the spin magnetization in the left-handed helix and that in the right-handed helix are opposite.

We have shown that in systems with helical structure, the orbital and spin magnetizations are induced by an electric current along the helical axis, using a simple tight-binding models. When an electric field is applied along the helical axis, the orbital magnetization is induced by the orbital magnetic moment on the Fermi surface, as a consequence of broken inversion symmetry. Similarly to the Berry curvature, the orbital magnetic moment is enhanced near band crossings. Therefore, when the Fermi energy lies near the band crossing, the orbital magnetization is enhanced as well. We have also shown that, in the helical crystal with spin-orbit interaction, a current induces the spin magnetization along the helical axis due to the radial spin texture. The absence of mirror symmetry allows to have the radial spin texture, which is completely different from Rashba systems with a tangential spin texture.

We have presented a toy model for a helical structure. From a symmetry viewpoint, the present effect of longitudinal current-induced magnetization appears for chiral crystals without inversion and mirror symmetries. Real helical crystals, such as Se and Te, also lack inversion and mirror symmetries. From symmetry consideration, we expect that orbital and spin magnetizations along the helical axis can be induced by an electric current in these crystals by doping carriers. Magnitudes of the induced magnetizations can be estimated from our results. The induced orbital magnetization scales with *e*^2^*E*_*z*_*τt*_1_/*ħ*^2^, which is about 150 gauss for *E*_*z*_ = 10^4^ V/m, *τ* = 10^−12^ s and *t*_1_ = 3 eV. In Fig. 2, the maximum value is 0.1 times the above scale, and this factor could be enhanced for more “helical” crystalline structure. On the other hand, the induced spin magnetization scales wtih *eE*_*z*_*τμ*_*B*_/*ħa*^2^, which is about 7 gauss for *E*_*z*_ = 10^4^ V/m, *τ* = 10^−12^ s and *a* = 0.5 nm. It is multiplied by a numerical factor in Fig. 3, approximately given by the ratio *λ*/*t*_1_. Thus, the size of the spin-orbit coupling limits the size of the current-induced spin magnetization. On the other hand, the orbital magnetization does not require spin-orbit coupling, and it can be enhanced by appropriate choice of materials with helical crystal structure. Te and Se consist of weakly coupled helices, and therefore they may have large current-induced orbital magnetization.

Our results also provide a new building block of spintronics and will pave the way to spintronics application of helical crystals. For example, consider a ferromagnet on a helical crystal. By injecting current into the helical crystal, the induced magnetization exerts a torque on the ferromagnet, thus leading to current-induced magnetization reversal.

## Methods

### Details of the calculation of current-induced spin magnetization

Within the Boltzmann approximation, the electric field *E*_*z*_ induces the spin magnetization as


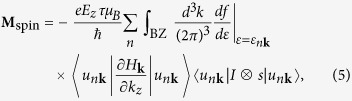


where *μ*_*B*_ is the Bohr magneton and the electron spin *g*-factor *g* ≈ 2. In the present model (4), for *λ* = 0 the spin magnetization always vanish, because of a cancellation between the contribution from (*k*_*x*_, *k*_*y*_, *k*_*z*_) and that from (−*k*_*x*_, −*k*_*y*_, *π*/*c* − *k*_*z*_). This cancellation can be avoided by adding either spin-dependent hoppings within the *xy* plane (for example, the *λ* term in Eq. (4)) or spin-independent hoppings between the neighboring layers (for example, the *t*_2_ term in Eq. (1)).

## Additional Information

**How to cite this article**: Yoda, T. *et al.* Current-induced Orbital and Spin Magnetizations in Crystals with Helical Structure. *Sci. Rep.* **5**, 12024; doi: 10.1038/srep12024 (2015).

## Figures and Tables

**Figure 1:**
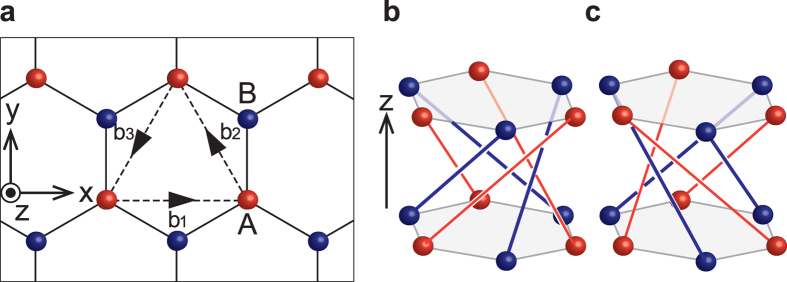
Helical lattice structure of the present model. **a** One layer of the model, forming a honeycomb lattice. Dashed arrows denote vectors **b**_1_, **b**_2_, and **b**_3_. **b** Hopping texture in the right-handed helix. **c** Hopping texture in the left-handed helix. Red (blue) lines denote hoppings between A (B) sites.

**Figure 2:**
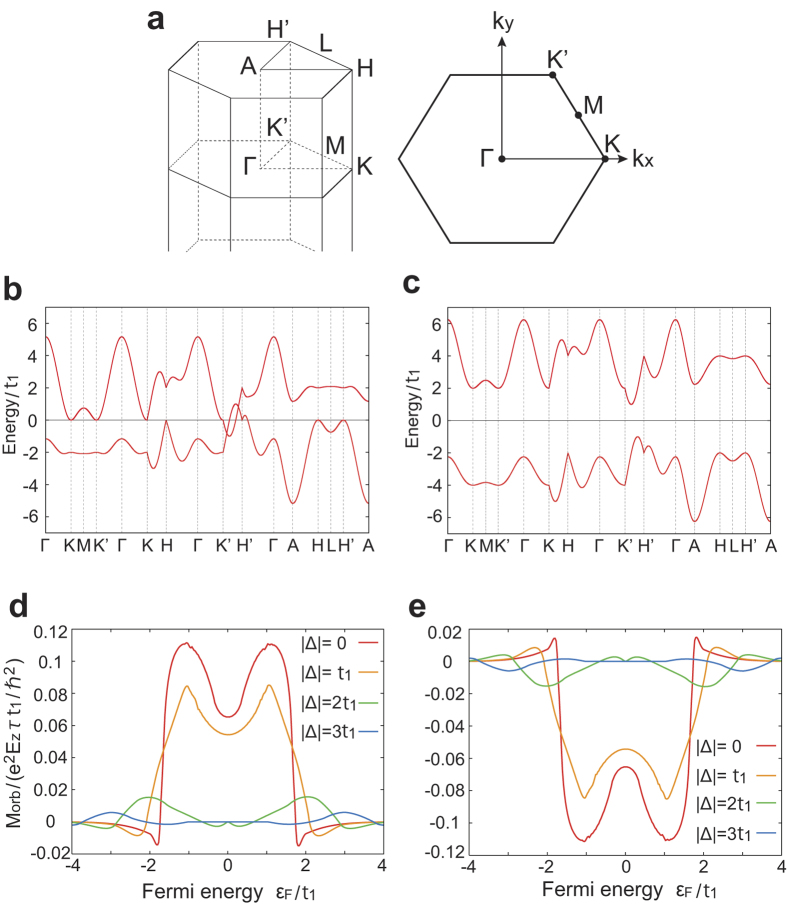
Current-induced orbital magnetization. **a** Brillouin zone of our model with high-symmetry points and that on *k*_*z*_ = 0. **b,c** Energy bands for the Hamiltonian (1) with *t*_2_ = *t*_1_/3, and **b** Δ = *t*_1_; **c** Δ = 3*t*_1_. The energy bands are indicated within 0 ≤ *k*_*z*_ ≤ *π*/*c*. **d,e** Orbital magnetization for several values of Δ in **d** the right-handed helix and **e** the left-handed helix. We set the parameters *t*_2_ = *t*_1_/3.

**Figure 3:**
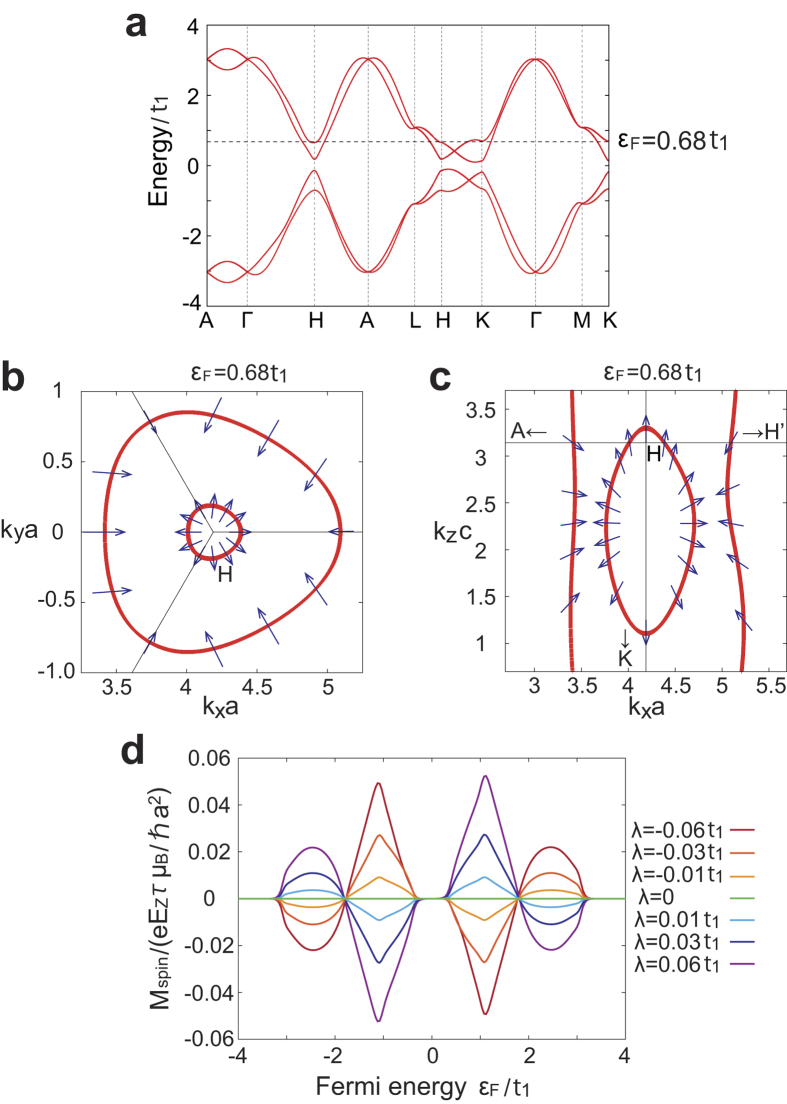
Current-induced spin magnetization. **a** Energy band for the Hamiltonian with *λ* = −0.06*t*_1_, *λ*_*xy*_ = *λ*_*z*_ = 0.05*t*_1_, and Δ = 0.4*t*_1_. Dashed line indicates *ε*_*F*_ = 0.68*t*_1_. **b**,**c** Fermi surface and spin texture. The parameters are the same as **a**. Arrows represent the spin projected onto each plane. The spin on the inner Fermi surface in **b** is drawn with three times larger scale. **d** Spin magnetization for several values of *λ*. The parameters are *λ*_*xy*_ = 0.05*t*_1_, *λ*_*z*_ = 0.05*t*_1_, and Δ = 0.4*t*_1_. We also set the temperature *T* = 0.03*t*_1_/*k*_B_.

## References

[b1] Spaldin N. A. & Fiebig M. The Renaissance of Magnetoelectric Multiferroics. Science 309, 391–392 (2005). 16020720 10.1126/science.1113357

[b2] Fiebig M. Revival of the magnetoelectric effect. Journal of Physics D: Applied Physics 38, R123 (2005).

[b3] Eerenstein M., Mathur N. D. & Scott J. F. Multiferroic and magnetoelectric materials. Nature 442, 759–765 (2006). 16915279 10.1038/nature05023

[b4] Cheong S.-W. & Mostovoy M. Multiferroics: a magnetic twist for ferroelectricity. Nature Materials 6, 13–20 (2007). 17199121 10.1038/nmat1804

[b5] Rößler U. K., Bogdanov A. N. & Pfleiderer C. Spontaneous skyrmion ground states in magnetic metals. Nature 442, 797–801 (2006). 16915285 10.1038/nature05056

[b6] Mühlbauer S. *et al.* Skyrmion Lattice in a Chiral Magnet. Science 323, 915–919 (2009). 19213914 10.1126/science.1166767

[b7] Fert A., Cros V. & Sampaio J. Skyrmions on the track. Nature Nanotechnology 8, 152–156 (2013). 10.1038/nnano.2013.29 23459548

[b8] Nagaosa N. & Tokura Y. Topological properties and dynamics of magnetic skyrmions. Nature Nanotechnology 8, 899–911 (2013). 10.1038/nnano.2013.243 24302027

[b9] Mangin S. *et al.* Current-induced magnetization reversal in nanopillars with perpendicular anisotropy. Nature Materials 5, 210–215 (2006).

[b10] Manchon A. & Zhang S. Theory of nonequilibrium intrinsic spin torque in a single nanomagnet. Phys. Rev. B 78, 212405 (2008).

[b11] Chernyshov A. *et al.* Evidence for reversible control of magnetization in a ferromagnetic material by means of spin-orbit magnetic field. Nature Physics 5, 656–659 (2009).

[b12] Miron I. M. *et al.* Current-driven spin torque induced by the Rashba effect in a ferromagnetic metal layer. Nature materials 9, 230–234 (2010). 20062047 10.1038/nmat2613

[b13] Haldane F. D. M. Model for a quantum Hall effect without Landau levels: Condensed-matter realization of the “parity anomaly”. Phys. Rev. Lett. 61, 2015–2018 (1988). 10038961 10.1103/PhysRevLett.61.2015

[b14] Xiao D., Shi J. & Niu Q. Berry phase correction to electron density of states in solids. Phys. Rev. Lett. 95, 137204 (2005). 16197171 10.1103/PhysRevLett.95.137204

[b15] Thonhauser T., Ceresoli D., Vanderbilt D. & Resta R. Orbital magnetization in periodic insulators. Phys. Rev. Lett. 95, 137205 (2005). 16197172 10.1103/PhysRevLett.95.137205

[b16] Ceresoli D., Thonhauser T., Vanderbilt D. & Resta R. Orbital magnetization in crystalline solids: Multi-band insulators, chern insulators, and metals. Phys. Rev. B 74, 024408 (2006).

[b17] Xiao D., Yao Y., Fang Z. & Niu Q. Berry-phase effect in anomalous thermoelectric transport. Phys. Rev. Lett. 97, 026603 (2006). 16907470 10.1103/PhysRevLett.97.026603

[b18] Shi J., Vignale G., Xiao D. & Niu Q. Quantum theory of orbital magnetization and its generalization to interacting systems. Phys. Rev. Lett. 99, 197202 (2007). 18233109 10.1103/PhysRevLett.99.197202

[b19] Xiao D., Chang M.-C. & Niu Q. Berry phase effects on electronic properties. Rev. Mod. Phys. 82, 1959–2007 (2010).

[b20] Resta R. Electrical polarization and orbital magnetization: the modern theories. Journal of Physics: Condensed Matter 22, 123201 (2010). 21389484 10.1088/0953-8984/22/12/123201

[b21] Chang M.-C. & Niu Q. Berry phase, hyperorbits, and the hofstadter spectrum: Semiclassical dynamics in magnetic bloch bands. Phys. Rev. B 53, 7010–7023 (1996). 10.1103/physrevb.53.7010 9982146

[b22] Sundaram G. & Niu Q. Wave-packet dynamics in slowly perturbed crystals: Gradient corrections and berry-phase effects. Phys. Rev. B 59, 14915–14925 (1999).

[b23] Hirayama M., Okugawa R., Ishibashi S., Murakami S. & Miyake T. Weyl node and spin texture in trigonal tellurium and selenium. *arXiv:1409.7517* 10.1103/PhysRevLett.114.206401 26047243

[b24] Edelstein V. M. Spin polarization of conduction electrons induced by electric current in two-dimensional asymmetric electron systems. Solid State Communications 73, 233–235 (1990).

[b25] Inoue J.-i., Bauer G. E. W. & Molenkamp L. W. Diffuse transport and spin accumulation in a Rashba two-dimensional electron gas. Phys. Rev. B 67, 033104 (2003).

[b26] Kato Y. K., Myers R. C., Gossard A. C. & Awschalom D. D. Current-induced spin polarization in strained semiconductors. Phys. Rev. Lett. 93, 176601 (2004). 15525098 10.1103/PhysRevLett.93.176601

[b27] Sih V. *et al.* Spatial imaging of the spin hall effect and current-induced polarization in two-dimensional electron gases. Nature Physics 1, 31–35 (2005).

